# Inability of *Dirofilaria immitis* infective larvae from mosquitoes fed on blood from microfilaremic dogs during low-dose and short-treatment regimens of doxycycline and ivermectin to complete normal development in heartworm naïve dogs

**DOI:** 10.1186/s13071-023-05704-5

**Published:** 2023-06-13

**Authors:** John Wilson McCall, Utami DiCosty, Abdelmoneim Mansour, Crystal Fricks, Scott McCall, Michael Timothy Dzimianski, Ben Carson

**Affiliations:** 1 TRS Labs, Inc., Athens, GA 30607 USA; 2 Department of Infectious Diseases, College of Veterinary Medicine, University of Georgia, Athens, GA 30602 USA

**Keywords:** *Dirofilaria immitis*, Mosquitoes, Migration, Development, Dogs, Doxycycline, Ivermectin, Blocking transmission

## Abstract

**Background:**

This study was conducted to determine whether heartworm infective larvae (L_3_) collected from mosquitoes fed on dogs during low-dose, short-treatment-regimen doxycycline and ivermectin could develop normally in dogs.

**Methods:**

Twelve Beagles in a separate study were infected with 10 pairs of adult male and female *Dirofilaria immitis* by IV transplantation and randomly allocated to three groups of four dogs. Starting on Day 0, Group 1 received doxycycline orally at 10 mg/kg sid for 30 days plus ivermectin (min., 6 mcg/kg) on Days 0 and 30; Group 2 received doxycycline orally at 10 mg/kg sid until individual dogs became microfilaria negative (72–98 doses) and ivermectin every other week for six to seven doses. These dogs served as microfilaremic blood donors for the current mosquito studies.

*Aedes aegypti* were allowed to feed on group-pooled blood samples from treated Groups 1-M and 2-M and untreated control Group 3-M on Days 22 (Study M-A) and 42 (Study M-C) and from Groups 1-M and 2-M on Day 29 (Study M-B) after treatment was started. From the Day 22 mosquito feeding, two dogs in Groups 1-M and 2-M and one dog in Group 3-M were given 50 L_3_ by SC inoculation. From the Day 29 feeding, two dogs in Groups 1-M and 2-M were given 50 L_3_. From the Day 42 feeding, two dogs in Group 1-M received 30 L_3_, while two dogs in Group 2-M and one dog in Group 3-M received 40 L_3_. All 14 dogs were necropsied for recovery and enumeration of adult heartworms 163–183 days PI.

**Results:**

None of the 12 dogs that received L_3_ from mosquitoes fed on blood from treated dogs 22, 29 or 42 days after treatment started had any adult heartworms at necropsy, while the two control dogs had a total of 26 and 43 heartworms, respectively.

**Conclusions:**

Treatment of microfilaremic dogs with doxycycline plus an ML, which later renders the L_3_ incapable of normal development in the animal host, widens the scope of the multimodal approach to heartworm prevention in reducing the spread of heartworm disease.

**Graphical Abstract:**

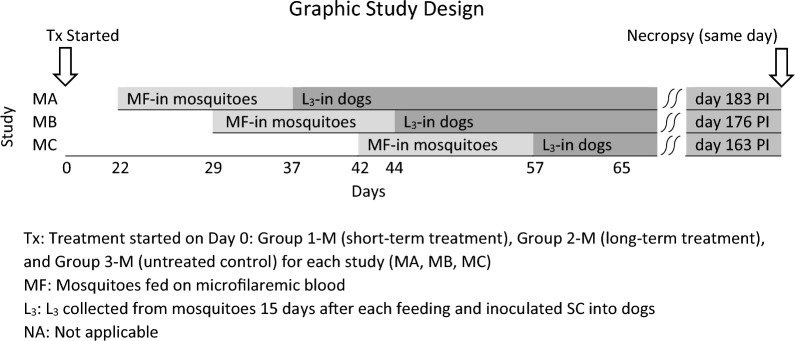

## Background

*Wolbachia* endosymbionts (*Wolbachia*) bacteria are present in every life cycle stage of *Dirofilaria immitis* and every other filarial species that harbors this bacteria, and they are needed for normal growth and development of these filarial parasites [1–3]. Tetracycline drugs have been shown to have adverse effects, ranging from sub-lethal to lethal, on various filarial life cycle stages (see [4] in this issue for further details), and there is general agreement that these antifilarial effects can be attributed to death of the *Wolbachia* because these antibiotics have no effect on *Wolbachia*-negative filarial species, such as *Acanthocheilonema viteae* [5, 6], and the antibacterial effects precede the antifilarial effects [3, 7, 8].

In an early study [9], treatment with doxycycline administered orally at 10 mg/kg for 1-month periods after experimental infection of dogs by SC inoculation of third-stage infective larvae (L_3_) was shown to be highly effective against the early developing stages of *D. immitis*. Treatment during the first month of infection (Days 0–29), which covers the time of infection with L_3_, the period of molting from L_3_ to L_4_ and the early period of infection with L_4_ [10], was 100% effective in preventing the establishment of infection. Treatment from Days 40–69, which covers the later time of infection with L_4_ and the molt from L_4_ to juveniles, was 98.4% effective. Treatment from Days 65–94, which covers most of the period when juvenile heartworms are arriving in the pulmonary arteries, was only 69.6% effective in killing juvenile heartworms but completely blocked subsequent production of microfilariae during the course of the study.

In a later study, *D. immitis* microfilaremic dogs were treated with doxycycline and ivermectin alone or together to test for microfilaricidal and adulticidal activity [11]. Doxycycline was administered orally at 10 mg/kg/day during Weeks 1–6, 10–11, 16–17, 22–25 and 28–33. Ivermectin was administered orally according to instructions on the label at a minimum dosage of 6 mcg/kg at weekly intervals until necropsy for recovery of adult worms at 36 weeks. All treatments started at Day 0. All dogs given both doxycycline and ivermectin were cleared of circulating mf by 12 weeks and the count never re-bounded, whereas some dogs given doxycycline or ivermectin alone still had a few mf at necropsy. Adulticidal efficacy was also higher in the doxycycline plus ivermectin group (78.3%) than in the groups administered either doxycycline (8.7%) or ivermectin (20.3%) alone. Also, when mosquitoes were fed blood collected from microfilaremic dogs infected with macrocyclic lactone (ML)-susceptible or -resistant isolates of *D. immitis* and treated with doxycycline alone, the L_3_ were normal in appearance and motility, but when injected SC into heartworm-naïve dogs would not complete normal growth and development with adult worms in the heart and associated vessels. In contrast, one of six L_3_ from mosquitoes fed on blood from ivermectin-treated dogs developed to the adult stage and was recovered from the heart and lungs at necropsy. These data strongly suggest that the combination of doxycycline and ivermectin had a synergistic effect against heartworms and that doxycycline, not ivermectin, was responsible for a more rapid antifilarial-growth-and-development effect on mf, which was later manifested by the lack of normal growth and development of the L_3_ in dogs.

As a follow-up to the above studies, another study was conducted to determine the effects of doxycycline administered orally, twice daily (bid) at 10 mg/kg for 30 days on heartworm embryogenesis, transmission, circulating mf and adult worms in microfilaremic dogs [12]. The study was designed to determine whether heartworm mf collected at later times after treatment would regain the ability to develop to L_3_ which could complete normal development in a dog. The study also yielded valuable data on the effects of treatment on mf, antigen levels and adult worms. Microfilaria levels gradually declined during the 12–13 months after treatment was initiated. Two of the five treated dogs were amicrofilaremic at necropsy, but three still had a few mf (13 or fewer/ml). Treated dogs had fewer live adult worms than controls at necropsy, but all of the remaining worms in the treated dogs were moribund. Regarding the antifilarial-growth-and-development effect, none of the L_3_ from mosquitoes fed on blood from treated dogs 73–77 or 161–164 days after treatment started and injected SC into heartworm-naïve dogs developed to adult worms.

The current mosquito study was designed to investigate whether L_3_ collected from mosquitoes fed on blood collected from dogs earlier, i.e. during low-dose and short- or long-treatment regimens of both doxycycline and ivermectin rather than after treatment with doxycycline only is stopped and/or mf counts are very low or negative, could develop normally in heartworm-naïve dogs.

## Methods

### General study design

#### Infection and treatment of microfilaremic donor dogs

Twelve adult Beagle dogs were infected with 10 pairs of adult male and female *D. immitis* (Berkeley isolate) by IV transplantation via a jugular vein [13]. After microfilaria counts were > 1,000 mf/ml, the dogs were randomly allocated to three groups of four dogs each. Starting on Day 0, Group 1 received doxycycline orally at approximately 10 mg/kg sid (once daily) for 30 days plus a heartworm preventive dose of ivermectin (min., 6 mcg/kg) on Days 0 and 30 (short-treatment regimen); Group 2 received doxycycline orally at approximately 10 mg/kg sid (once daily) until each dog was mf-negative (range, 72–98 doses) and a preventive dose of ivermectin every other week for a total of 6–7 doses (long-treatment regimen), Group 3 served as the untreated controls. Microfilaria counts (modified Knott test) were done prior to the first day of treatment, at 1- to 2-week intervals during the first 4 months and then monthly thereafter. These treated (8) and untreated control (4) dogs served as microfilaremic blood donors for this study. For further details on these dogs, see data published elsewhere in this issue of the journal [4].

#### ***SC inoculation of heartworm-naïve dogs with L***_***3***_*** collected from mosquitoes and necropsy of dogs***

Female *Aedes aegypti* were allowed to feed on group-pooled blood samples from the treated and/or untreated control groups on Days 22 (Study M-A), 29 (Study M-B) and/or 42 (Study M-C) as follows: from Groups 1-M (short-treatment regimen), 2-M (long-treatment regimen) and 3-M (untreated control) on Days 22 and 42 and from Groups 1-M and 2-M on Day 29 (see Table 1). L_3_ were collected 15 days after each of the three mosquito infection days. Microfilaremia levels in all of the treated groups and untreated controls were sufficiently high to obtain enough heartworm L_3_ to infect all of the total of 14 dogs for Studies M-A, M-B and M-C with 30–50 L_3_ per dog (see Table 1). For Study M-A, a total of five dogs were inoculated SC with 50 L_3_ collected from mosquitoes that had fed on blood from treated dogs in Group 1-M (2 dogs), treated dogs in Group 2-M (2 dogs) and untreated control dogs in Group 3-M (1 dog) 22 days after treatment started, and the dogs were necropsied for recovery and counting of adult heartworms at 183 days post-infection (PI). For Study M-B, four dogs were inoculated SC with 50 L_3_ collected from mosquitoes that had fed on blood from treated dogs in Group 1-M (2 dogs) and Group 2-M (2 dogs) 29 days after treatment started. An untreated control for this study was not included. These four dogs were necropsied for recovery and counting of adult heartworms at 176 days PI. Smaller inocula (30–40 L_3_) were used for Study M-C because of a lower recovery of L_3_. For Study M-C, five dogs were inoculated SC with either 30 L_3_ collected from mosquitoes that had fed on blood from treated Group 1-M (2 dogs) or 40 L_3_ collected from mosquitoes that had fed on blood from treated Group 2-M (2 dogs) or the untreated control Group 3-M (1 dog) 42 days after treatment started. These five dogs were necropsied for recovery of adult heartworms at 163 days PI. As shown above, inoculation days for the studies were different, but all 14 dogs were humanely killed and necropsied for recovery and enumeration of adult heartworms on the same day, i.e. 163, 176 or 183 days PI.

**Table 1:** *Dirofilaria immitis* recovery for dogs infected with L_3_ from mosquitoes fed on blood from dogs treated with doxycycline plus ivermectin and untreated controls

Study	Dog	Group	Study day mosquitoes infected	No. L3 per dog	Necropsy days Pl	Male	Female	Total
No. adult heartworms								
M-A	UIAQ	1-M^a^	22	50	183	0	0	0
	9285	1-M	22	50	183	0	0	0
	9284	2-M	22	50	183	0	0	0
	147	2-M^b^	22	50	183	0	0	0
	175	3-M^c^	22	50	183	18	25	43
M-B	208	1-M	29	50	176	0	0	0
	209	1-M	29	50	176	0	0	0
	210	2-M	29	50	176	0	0	0
	211	2-M	29	50	176	0	0	0
M-C	140	1-M	42	30	163	0	0	0
	164	1-M	42	30	163	0	0	0
	SIBO	2-M	42	40	163	0	0	0
	144	2-M	42	40	163	0	0	0
	151	3-M	42	40	163	12	14	26

^a^Short-term treatment: single, daily oral doses of doxycycline administered at 10 mg/kg for 30 days starting on Day 0 and monthly prophylactic doses of ivermectin (min., 6 mcg/kg) administered on Day 0 and Day 30

^b^Long-term treatment: single, daily oral doses of doxycycline administered at 10 mg/kg (72–98 days) and bi-weekly doses of prophylactic doses of ivermectin (min. dose, 6 mcg/kg) administered orally every 2 weeks (6–7 doses) until individual dogs became amicrofilaremic

^c^Untreated controls

#### Mosquito strain and heartworm isolate

In 1972, the University of Georgia (UGA), USA, obtained the Black-eyed Liverpool strain of *Ae. aegypti* used in this study from Prof. W.W. Macdonald in the Department of Parasitology and Entomology at the Liverpool School of Tropical Medicine (UK), who had obtained it from West Africa in 1962 [14]. When the high susceptibility to filarial parasites, including *D. immitis*, was established as an inherited character, the mode and pattern of inheritance were investigated and reported [15]. TRS Labs, Inc., obtained the strain from UGA in 1980. During the 50 years the mosquitoes were maintained at UGA and then TRS, it is estimated the strain was maintained through a total of 2009 generations. Moreover, during the 42 years that the strain was maintained at both UGA and TRS, both laboratory colonies were refreshed with eggs from the other colony, i.e. from TRS to UGA and from UGA to TRS.

The Berkeley isolate of *D. immitis* was used in this study. The isolate was obtained by TRS from Berkeley County, SC, USA, in April 2014 and was validated by testing positive for heartworm microfilariae and antigen and by worm recovery at necropsy in December 2014. It is known to be susceptible to ML heartworm preventives [16].

#### Animals and animal management

Fourteen purpose-bred male (10) and female (4) Beagles from a commercial supplier were used in this study. They ranged in age from 4.5 to 11.5 months on the day of infection and ranged from 6.7 to 13.3 kg in body weight 1–2 months after infection. They were born and raised indoors in mosquito-proof facilities and were not treated with any heartworm preventive drugs prior to the start of this study. They had negative test results for microfilariae (modified Knott test) and adult heartworm antigen (DiroCHEK^™^ Heartworm Antigen Test Kit, Zoetis, Inc., Kalamazoo, MI, USA) and were randomly allocated by a table of random numbers to three groups of four dogs each just prior to infection of the first set of dogs. The dogs were housed individually in 4 × 5-foot kennels during dosing. Thereafter, they were pair-housed by gender within groups, i.e. with access to their mate’s kennel. This study was approved by the TRS Institutional Animal Care and Use Committee prior to initiation of the study, and the dogs received humane care, with at least a once daily health observation, throughout the study.

#### Study drugs

Doxycycline hyclate was administered as one 100-mg tablet or two 50-mg capsules (Harris Pharmaceuticals) orally once daily to achieve a dosage of approximately 10 mg/kg/day. The doxycycline daily dose range for Group 1 treated dogs was 9.1–10.9 mg/kg, and the daily dose range for Group 2 treated dogs was 9.6–15.4 mg/kg.

Ivermectin was administered orally as 68 mcg chewables (Iverhart^®^, Virbac, Ft. Worth, Texas) to achieve a minimum dose of 6.0 mcg/kg ivermectin. Each treated dog received one ivermectin chewable per dose. The monthly dose range for Group 1 treated dogs was 6.2–7.4 mcg/kg, and the bi-weekly dose range for Group 2 treated dogs was 6.5–10.5 mcg/kg.

### Statistical analysis

A statistical analysis of the data was not done, as all of the dogs in all of the treated groups were negative for adult heartworms at necropsy and all of the untreated control dogs had heartworms.

## Results

### Recovery of adult heartworms at necropsy

As shown in Table 1, none of the 12 dogs inoculated with L_3_ collected from mosquitoes that had fed on blood from treated dogs (Groups 1-M and 2-M) 22, 29 or 42 days after treatment started had heartworms, while the untreated control dog (Group 3-M) in Study M-A had a total of 43 adult heartworms and the untreated control dog (Group 3-M) in Study M-C had a total of 26 adult heartworms.

## Discussion

For heartworm adulticidal therapy, the American Heartworm Society currently recommends the initiation of daily, oral doses of doxycycline administered at 10 mg/kg bid for 28 days along with a ML heartworm preventive 2 months before the first injection of the three-injection protocol for melarsomine are given [17, 18]. While the doxycycline plus ML treatment will eventually clear microfilariae from the blood, some microfilariae may persist for up to 3 weeks following administration of high-dose moxidectin plus doxycycline [19] and up to 5.6 months following initiation of twice daily doses (bid) of 10 mg/kg of doxycycline for 30 days and 6 monthly prophylactic doses of ivermectin [20] or up to 8 months following single daily doses (sid) of 10 mg/kg of doxycycline for 72–98 days and 6–7 bi-weekly prophylactic doses of ivermectin [4] (in this issue). Considering that circulating mf are present for weeks to months after doxycycline plus ML treatment is started, the question arises as to whether these lingering mf are capable of infecting susceptible mosquito vectors and, if so, whether the L_3_ from these mosquitoes will develop normally and migrate to the heart and pulmonary arteries of dogs after transmission. Earlier work in this laboratory demonstrated that L_3_ collected from mosquitoes fed on blood from dogs 4 days or later after 6 weeks of doxycycline administered orally at 10 m/kg sid with no ML [11] or 43–134 days after 30 days of doxycycline administered orally at 10 mg/kg bid for 30 days with no ML [12] were not capable of developing and migrating to the heart and pulmonary arteries of heartworm-naïve dogs.

The present study demonstrates the high level of growth- and development-suppressing activity against heartworm microfilariae of single, low daily doses of doxycycline for relatively short periods plus only 1 monthly or several bi-weekly doses of ivermectin. No adult heartworms were recovered from any of the 12 dogs that had been treated with doxycycline for 22, 29 or 42 days and had also been given only a single dose of ivermectin or two doses 1 month apart, or three doses at bi-weekly intervals. Furthermore, this low dose of doxycycline (10 mg/kg) administered sid for only 22 days, along with a single dose of ivermectin on Day 0, was sufficient to prohibit the development of L_3_ to adults in the heartworm-naïve dogs.

The results of the current study also expand the range of this 100% heartworm-disease-blocking effect of doxycycline plus ivermectin to as early as 22 days after initiation of a single, low daily dose of doxycycline at 10 mg/kg sid plus a single prophylactic dose of ivermectin administered on Day 0. A follow-up study is currently underway in this laboratory to test this effect on heartworm mf in microfilaremic dogs during daily doses of 5.0, 7.5 and 10.0 mg/kg of doxycycline bid (for 28 days) and a prophylactic dose of ivermectin at Day 0, with microfilaremic blood collected from these dogs being fed to mosquitoes 14 and 21 days after dosing is started. These data will be published later.

The conventional approach to heartworm disease prevention is oral, topical or parenteral administration of ML heartworm preventive products. However, the treatment of domestic microfilaremic dogs (and domestic cats) with doxycycline plus an ML, which later renders the L_3_ incapable of normal development in the animal host, widens the scope of the multimodal approach to reducing the spread of heartworm in domestic and wild dogs and cats, ferrets and susceptible exotic animals. In addition, this approach includes vector mosquito control using conventional methods such as insecticide application, biological control, drainage of standing water and direct treatment of animals, particularly dogs, by topical application of repellent ectoparasiticides to repel and kill infected or uninfected adult mosquitoes before biting and transmission of mf to mosquitoes or L_3_ to dogs occur [21, 22]. For some time, it has been known that topical administration of insecticides is effective in reducing vector mosquito populations [25–36]. It has only recently been shown that oral administration of some isoxazalines has a similar effect [23] (in this issue) [24]. Effectively reducing populations of heartworm-infected and -uninfected mosquitoes, along with a high owner compliance in the administration of conventional ML preventive drugs, should greatly diminish the spread of heartworm disease.

## Conclusion

The data from this study strongly support the inclusion of doxycycline and an ML in the American Heartworm Society recommended heartworm adulticide protocol, as it adds the significant epidemiological benefit of blocking the normal development of heartworm L_3_, even ML-resistant biotypes [12], after they are transmitted under natural conditions to the animal host.

## Data Availability

Not applicable.
